# Heme Oxygenase‐1 Differentially Controls Pigmentation in Physiological and Pathological Melanogenesis

**DOI:** 10.1111/pcmr.70105

**Published:** 2026-07-06

**Authors:** Agnieszka Seretny, Grażyna Jamróg, Jacek Stępniewski, Maciej Cieśla, Witold Nowak, Rościsław Krutyhołowa, Martin Petrovic, Anna Tejchman‐Skrzyszewska, Milena Mazan, Halina Waś, Wolfgang Weninger, Matthias Farlik, Alicja Józkowicz, Anna Kusienicka

**Affiliations:** ^1^ Department of Medical Biotechnology, Faculty of Biochemistry, Biophysics, and Biotechnology Jagiellonian University Krakow Poland; ^2^ Department of Dermatology Medical University of Vienna Vienna Austria; ^3^ Department of Molecular Biology National Institute of Geriatrics, Rheumatology and Rehabilitation Warsaw Poland

**Keywords:** heme oxygenase‐1, melanocytes, melanogenesis, melanoma, melanosome trafficking, pigmentation, tyrosinase

## Abstract

Melanogenesis is a defining feature of melanocyte biology and influences melanoma pathogenesis; however, tumor‐specific regulators of this process remain incompletely understood. Here, we investigate how heme oxygenase‐1 (HO‐1) regulates pigmentation in malignant and homeostatic contexts. Using B16‐F10 melanoma cells engineered to express varying levels of HO‐1, we show that HO‐1 abundance correlates with pigmentation intensity and tyrosinase activity, without affecting transcription of core melanogenesis genes. Under pigment‐inducing conditions, HO‐1 inversely regulates intracellular melanin retention and pigment export, with inhibition of melanosome transfer confirming a role for HO‐1 in melanosome trafficking and secretion. Co‐culture experiments reveal that stromal HO‐1 promotes melanoma pigmentation through paracrine effects on tyrosinase expression. Transcriptomic analyses of human melanoma datasets show that *HMOX1* expression correlates with pathways related to melanosome acidification, copper homeostasis, and lysosomal transport rather than classical melanogenic programs. In contrast, HO‐1 is dispensable in the non‐malignant melanocytic conditions tested here—murine iPSC‐derived melanocytes during differentiation and Melan‐A melanocytes. Together, these findings identify HO‐1 as a context‐dependent regulator of melanoma pigmentation and a potential selective target for modulating pigmentation‐dependent tumor traits.

## Highlights

**Research Highlights:**

- HO‐1 acts as a context‐dependent regulator of melanoma pigmentation.
- HO‐1 promotes melanoma pigmentation by enhancing tyrosinase activity.
- HO‐1 deficiency shifts pigment handling toward increased melanin export.
- HO‐1 loss does not alter pigmentation in non‐malignant melanocytes.

**Significance:**

Pigmentation contributes to melanoma progression, yet the mechanisms that distinguish physiological melanogenesis from melanoma‐associated pigmentation remain incompletely defined. This study identifies heme oxygenase‐1 (HO‐1) as a context‐dependent regulator of pigmentation that operates robustly in the melanoma model but was not detected in the non‐malignant melanocytic models tested (murine iPSC‐derived melanocytes and Melan‐A melanocytes), influencing melanin synthesis and melanosome‐associated processes. These findings refine our understanding of pigmentation control in melanoma and highlight HO‐1 as a potential modulator of pigment‐associated tumor traits.

## Graphical abstract

HO‐1 acts as a context‐dependent regulator of pigmentation, enhancing tyrosinase activity and melanin accumulation in melanoma cells while having no detectable effect on pigmentation in the non‐malignant melanocyte models tested.

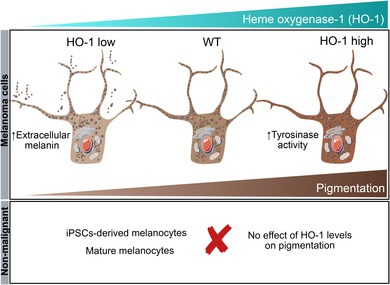

## 1 Introduction

Melanogenesis is a tightly regulated process in melanocytes, occurring within specialized organelles known as melanosomes. It involves the synthesis of melanin and the subsequent transfer of melanosomes to keratinocytes (Yamaguchi et al. 2007; D'Mello et al. 2016). By absorbing UV radiation and scavenging free radicals, melanin plays a cytoprotective role in the skin (D'Mello et al. 2016). However, this protective function also renders melanocytes particularly susceptible to oxidative stress, which arises both from UV‐induced damage and from melanin synthesis—processes that generate reactive oxygen species (ROS) and hydrogen peroxide (H_2_O_2_) (Denat et al. 2014). This oxidative burden may ultimately disrupt melanocyte homeostasis and contribute to the development of melanoma, the most aggressive form of skin cancer (Emanuelli et al. 2022). To counteract oxidative damage and maintain cellular redox balance, melanocytes employ antioxidant defense mechanisms. One such mechanism involves the induction of heme oxygenase‐1 (HO‐1), an enzyme that degrades heme into biliverdin, ferrous ions (Fe^2+^), and carbon monoxide (CO) (Was et al. 2010).

In melanocytes, UVA radiation and H_2_O_2_ induce HO‐1 expression via the redox‐sensitive transcription factor NRF2, positioning HO‐1 as a central component of the melanocyte stress‐defense program (Elassiuty et al. 2011; Marrot et al. 2005; Jian et al. 2011). Heme degradation by HO‐1 alters intracellular iron availability, which may enhance melanogenesis, since Fe^2+^ uniquely stimulates the activity of tyrosinase (Palumbo et al. 1985), the rate‐limiting enzyme in melanin synthesis. Furthermore, HO‐1 activates β‐catenin signaling, promoting melanocyte survival and upregulating microphthalmia‐associated transcription factor (MITF), the master regulator of melanogenesis and melanosome biogenesis (Kim et al. 2017). Through these pathways, HO‐1 could potentially enhance pigmentation. Yet, as mild oxidative stress can stimulate melanin synthesis (Tang et al. 2012), HO‐1 activity might conversely attenuate this melanogenic response. Consequently, the role of HO‐1 in melanogenesis and its broader impact on pigmentation of melanocytes and melanoma cells is still poorly understood.

Melanoma cells exhibit remarkable phenotypic plasticity, allowing dynamic transitions between distinct transcriptional states. Recently, we have shown that a transient subpopulation of slowly dividing murine melanoma cells, capable of initiating and sustaining tumor growth, displays reduced expression of melanogenesis‐associated genes (Kusienicka et al. 2023). Consistently, melanoma cells can reversibly switch between a MITF^high^ melanocytic (proliferative) and a MITF^low^ mesenchymal‐like (invasive) phenotype (Ennen et al. 2017). These transcriptional and functional states are tightly linked to melanin content and tumor behavior. However, the prognostic value of pigmentation in melanoma remains context‐ and stage‐dependent (Brożyna et al. 2013, 2016). While high intracellular melanin can reduce cell motility and invasiveness, secreted melanosomes may promote tumor progression by reprogramming dermal fibroblasts into cancer‐associated fibroblasts (Sarna et al. 2019, 2018; Dror et al. 2016). These opposing effects of melanin highlight the complexity of melanogenesis in melanoma progression and underscore the need to define its molecular regulators.

Previously, our group demonstrated that HO‐1 influences melanoma development by promoting tumor growth while reducing tumor‐initiating capacity (Was et al. 2006; Kusienicka et al. 2022). Building on these observations, we examined the effects of HO‐1 on melanogenesis in melanoma cells. Furthermore, we investigated whether the impact of HO‐1 on melanogenesis is context‐dependent by assessing its role in both pathological and physiological settings. Our findings indicate that both intrinsic and extrinsic HO‐1 expression enhance pigmentation in melanoma cells. In contrast, HO‐1 does not appear to affect pigmentation during physiological melanocyte differentiation or in mature melanocytes. Taken together, these results reveal a context‐specific role of HO‐1 and underscore its contribution to melanoma pathology.

## 2 Material and Methods

### 2.1 Cell Cultures

All cells were maintained under standard conditions (37°C, 5% CO_2_, 95% humidity). The source of cell lines used in the study and cell culture media are listed in the Table S1.

### 2.2 Isolation of Murine Mesenchymal Stromal Cells

MSCs were isolated from femurs and tibiae of C57BL/6J × FVB *Hmox1* ^*+/+*^ and *Hmox1* ^*−/−*^ mice (four mice per group) constitutively expressing GFP, following a published protocol (Nowak et al. 2018).

### 2.3 Isolation of Murine Tail‐Tip Fibroblasts

Tail tips from C57BL/6J × FVB *Hmox1* ^*+/+*^ and *Hmox1* ^*−/−*^ mice were disinfected in 70% ethanol, rinsed in PBS, skinned, minced, and digested in collagenase II (1.5 mg/mL, Gibco) for 2 h at 37°C with intermittent vortexing. Digests were centrifuged (300 *g*, 5 min), and the supernatants were transferred and recentrifuged under the same conditions. Pelleted cells were plated in culture medium on 0.1% gelatin‐coated dishes (Sigma‐Aldrich).

### 2.4 Generation of Cell Lines With Distinct HO‐1 Levels

B16‐F10 cells were transduced with retroviral vectors (RVs) carrying the *Hmox1* expression cassette driven by CMV promoter in the pBABE‐Puro backbone, as described previously (Kusienicka et al. 2022). HO‐1 knockdown in B16‐F10 and Melan‐A cells was achieved by RVs transduction with *Hmox1* mouse shRNA constructs (OriGENE, TF500966). For RVs production, Phoenix‐Eco cells were transfected with four different shHO‐1 plasmids and one scrambled control (Table S1), together with the M13 packaging plasmid (Addgene), using polyethyleneimine (MW 25000, Polysciences Inc.) according to the manufacturer's protocol. Next day, the medium was replaced with DMEM complete medium (CM) with 25 mM HEPES and after another 24 h RV‐containing media were collected, filtered through a 0.45 μm PVDF filter (Merck), and mixed in 1:1 with fresh culture medium containing polybrene (8 μg/mL; Sigma‐Aldrich). Target cells were exposed to the RVs for 48 h, selected with 1 μg/mL puromycin (Sigma‐Aldrich), and assessed for HO‐1 levels (Figure S1 and Figure 8a).

### 2.5 Generation of Induced Pluripotent Stem Cells (iPSCs) and Differentiation Toward Melanocytes

The detailed iPSCs methods are described in Data S1.

### 2.6 Cell Counting

Cell viability and total cell number were assessed using the Muse Count & Viability Kit (Millipore). At the indicated time points, cells were trypsinized, stained according to the manufacturer's instructions, and analyzed with the Muse Cell Analyzer (Millipore).

### 2.7 Co‐Culture of MSCs and B16‐F10‐Luc Cells

Previously established B16‐F10 cells (B16‐F10‐Luc) stably expressing the luciferase (Luc) transgene (Kusienicka et al. 2022) were seeded (50 cells/well, 24‐well plates) on a confluent monolayer of MSCs. Following the addition of luciferin (TriMen Chemicals), luminescence of B16‐F10‐Luc cells was monitored with the IVIS Lumina system for up to 6 days.

### 2.8 Fibrin Bead Assay

B16‐F10 cells were incubated with latex beads (Sigma‐Aldrich) at a 400:1 ratio in 1.5 mL RPMI 1640 medium in FACS tubes (37°C, 4 h, with gentle mixing every 20 min). The cell‐coated beads were transferred to T‐25 culture flasks and left overnight. The following day, beads were transferred to 15 mL conical tubes, allowed to sediment, and resuspended in EGM‐2 culture medium (Lonza). After counting, beads were resuspended in fibrinogen solution (2 mg/mL; Sigma‐Aldrich) containing aprotinin (0.15 U/mL; Sigma‐Aldrich) at a density of 500 beads/mL. Thrombin (0.625 U/mL, Sigma‐Aldrich) was added to each well, followed by 0.5 mL of the fibrin‐bead suspension and mixing. After a 15‐min incubation at 37°C, MSCs isolated from *Hmox1* ^+/+^ and *Hmox1* ^−/−^ mice were seeded (5000 cells/well) on top in 1 mL of EGM‐2 culture medium. Proliferation and migration of melanoma cells were monitored microscopically and quantified using ImageJ software.

### 2.9 In Vitro Tyrosinase Activity Analysis

Tyrosinase activity was assessed based on its catecholase function, measuring the oxidation of L‐DOPA to *o*‐dopaquinone (Fan et al. 2021; Nakazawa et al. 1985). Protein lysates were prepared in PBS containing 1% Triton X‐100 and a protease inhibitor (Roche). Enzyme activity was measured spectrophotometrically by monitoring the *o*‐dopaquinone formation, indicated by an increase in absorbance at 475 nm. Reactions contained 50 μg of protein and the substrate, L‐3,4‐dihydroxyphenylalanine (L‐DOPA; 2 mg/mL, Sigma‐Aldrich). Measurements were performed at 37°C for 180 min using a TECAN Infinite M200 microplate reader.

### 2.10 RNA Isolation and qRT‐PCR

Total RNA was extracted using Fenozol (A&A Biotechnology) with standard phenol‐chloroform separation (Florczyk‐Soluch et al. 2018). RNA quality was assessed by Nanodrop. cDNA was synthesized using the RevertAid RT Kit (Thermo Fisher Scientific). qRT‐PCR was performed with 10 ng cDNA and SYBR Green JumpStart Taq ReadyMix (Sigma‐Aldrich) on a StepOnePlus system (Applied Biosystems). Primer sequences are listed in Table S1.

### 2.11 Western Blotting

Proteins were isolated using RIPA lysis buffer supplemented with protease inhibitors (Roche). Proteins were separated by SDS‐PAGE and transferred onto nitrocellulose membranes and probed with primary and HRP‐conjugated secondary antibodies (Table S1). Protein bands were visualized using the Immobilon Western HRP Chemiluminescence Substrate (Millipore). Signals were detected with the ChemiDoc MP Imaging System and Image Lab 6.0 software (Bio‐Rad) or developed on X‐ray films.

### 2.12 FACS Analysis of Highly Pigmented Melanoma Cells

After 4 days of melanogenesis induction in DMEM CM, cells were trypsinized, centrifuged (300 *g*, 5 min), resuspended in PBS, and analyzed by flow cytometry to assess changes in cell size and granularity. Data were acquired on a BD LSR‐Fortessa flow cytometer. To inhibit melanosome transfer, niacinamide (10 μM; Sigma‐Aldrich) was added to DMEM CM during melanogenesis induction.

### 2.13 Intracellular Melanin Content

Cells were harvested after 4 days of melanogenesis induction in DMEM CM and lysed in 1 M NaOH for 60 min at 80°C (Chung et al. 2019). Lysates were centrifuged (250 *g*, 5 min) and absorbance of supernatants was measured at 405 nm using a TECAN Infinite M200 microplate reader.

### 2.14 Intracellular ROS

B16‐F10 cells were exposed to H_2_O_2_ (100 μM, 30 min), then incubated with CellROX Orange (5 μM, 30 min, 37°C), washed with PBS, stained with DAPI (0.2 μg/mL), and analyzed for MFI on a BD LSR‐Fortessa cytometer.

### 2.15 ATP Production

ATP production was measured using the Seahorse XF Cell Mito Stress Test (Agilent Technologies) according to the manufacturer's instructions. One day prior to the assay, cells were seeded in a Seahorse XF Cell Culture Microplate (Agilent Technologies) at a density of 10,000 cells per well. The assay was performed in DMEM supplemented with 1 mM pyruvate, 2 mM glutamine, and 10 mM glucose (all from Sigma‐Aldrich). The test was run using an Agilent Seahorse analyzer (XF96), and data were analyzed with Wave Software.

### 2.16 Transcriptomic Data Analysis

Normalized RNA‐seq data from the TCGA Skin Cutaneous Melanoma (SKCM) and Uveal Melanoma (UVM) cohorts (Firehose Legacy) were obtained via cBioPortal (https://www.cbioportal.org/). Pigmentation‐related gene sets were curated from the literature and supplemented with additional expert selections (Table S2). Spearman correlation coefficients were calculated between the expression of HMOX1 and individual genes within the defined gene set.

### 2.17 Experimental Schematics and Graphical Abstract

Graphs were created using modified images downloaded from Servier Medical Art (https://smart.servier.com/), and Bioicons portal (https://bioicons.com/) licensed under CC BY 3.0 and 4.0 and (https://creativecommons.org/licenses/).

### 2.18 Statistical Analysis

Data were analyzed using GraphPad Prism 10 or Microsoft Excel. Statistical significance was assessed with a two‐tailed Student's *t*‐test (for comparisons between two groups), or one‐way/two‐way ANOVA with Tukey's posttest (for three or more groups). Results were considered statistically significant at *p* < 0.05.

## 3 Results

### 3.1 HO‐1 Levels Correlate With Pigmentation in Melanoma Cells

**FIGURE 1:**
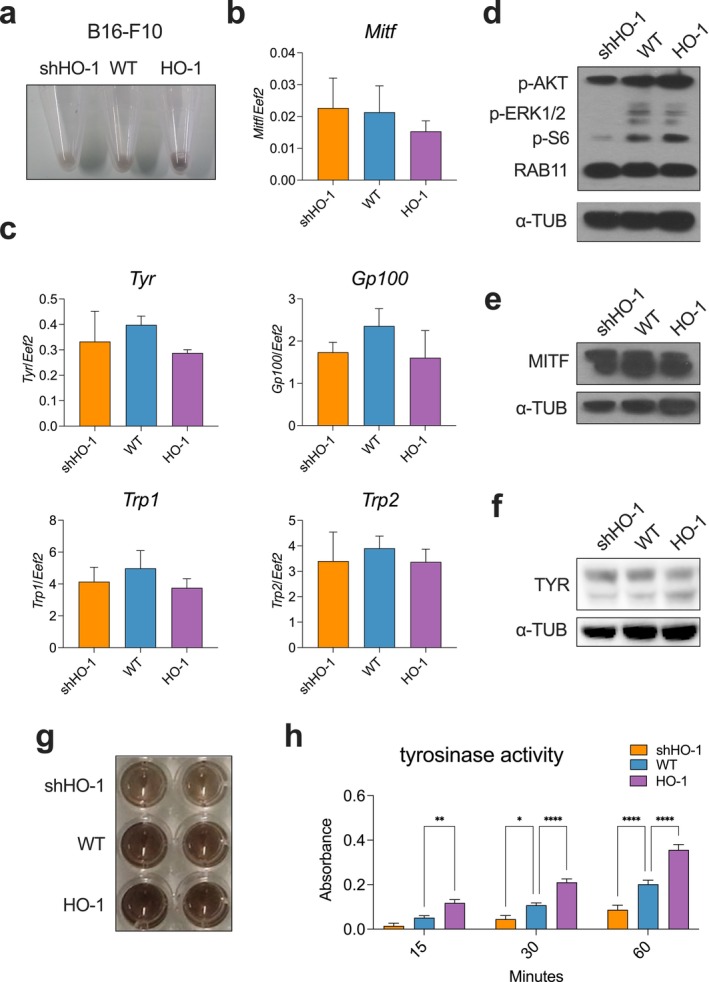
HO‐1 levels determine pigmentation and melanogenesis in B16‐F10 melanoma cells. (a) Representative pellets from B16‐F10 cell lines with different HO‐1 levels (decreased – shHO‐1, normal – WT, increased – HO‐1). (b) qRT‐PCR analysis of *Mitf* gene (each bar represents mean + SEM, *n* = 4). (c) qRT‐PCR analysis of MITF target genes: *Gp100*, *Tyr*, *Trp1*, and *Trp2* (each bar represents mean + SEM, *n* = 4). *Eef2* was used as a reference gene. (d) Western blot analysis of phosphorylation of AKT, ERK1/2, and S6 ribosomal protein. RAB11 was used as a loading control (representative blot, *n* = 2). (e) Western blot analysis of MITF protein levels (representative blot, *n* = 3). (f) Western blot analysis of tyrosinase protein levels (representative blot, *n* = 3). Tubulin was used as a loading control in e,f. (g) Representative picture of tyrosinase activity assay. (h) Absorbance measurement of tyrosinase activity measured at 475 nm 15, 30, and 60 min after addition of L‐DOPA substrate (each bar represents mean + SEM, *n* = 3; *p < 0.05, **p < 0.01, ****p < 0.0001).

To investigate the effects of HO‐1 on melanogenesis in melanoma cells, we generated murine melanoma B16‐F10 cell lines with three distinct HO‐1 expression levels: silenced HO‐1 (shHO‐1), control wild‐type (WT), and HO‐1 overexpression (HO‐1) (Figure S1). HO‐1 expression correlated with pigmentation intensity, with HO‐1‐silenced cells appearing lighter and HO‐1‐overexpressing cells darker than controls after centrifugation (Figure 1a). To identify the underlying mechanisms, we first examined the expression of *Mitf*, the master regulator of melanogenesis genes (Kawakami and Fisher 2017). *Mitf* mRNA levels remained stable irrespective of HO‐1 (Figure 1b). Accordingly, the expression of MITF target genes (*Tyr*, *Gp100*, *Trp1*, and *Trp2*) also remained unchanged (Figure 1c). Because regulation of MITF activity is complex, ranging from transcription to posttranslational modification, we next analyzed selected upstream pathways, including Mitogen Activated Kinases (MAPKs) and ribosomal S kinases (RSKs) (Liu et al. 2010). We found that HO‐1‐depleted melanoma cells show reduced phosphorylation of ERK1/2 and ribosomal protein S6 (Ser235/236); however, MITF protein levels remained unaffected (Figure 1d,e). Moreover, the rate‐limiting enzyme of melanin synthesis, tyrosinase, showed no significant differences in protein level among the cell lines (Figure 1f).

As tyrosinase expression levels do not necessarily correlate with enzymatic activity (D'Mello et al. 2016; Chang 2009), we measured *o*‐dopaquinone formation from L‐DOPA in cell lysates as a proxy for tyrosinase activity (Figure 1g). Interestingly, tyrosinase enzymatic activity correlated with HO‐1 levels, being lowest in shHO‐1 cells and highest in HO‐1‐overexpressing cells (Figure 1h). These findings indicate that HO‐1 promotes pigmentation in melanoma cells by enhancing tyrosinase activity rather than through transcriptional or translational regulation of melanogenesis genes.

### 3.2 HO‐1 Alters Redox Responses Without Basal ROS Shift

**FIGURE 2:**
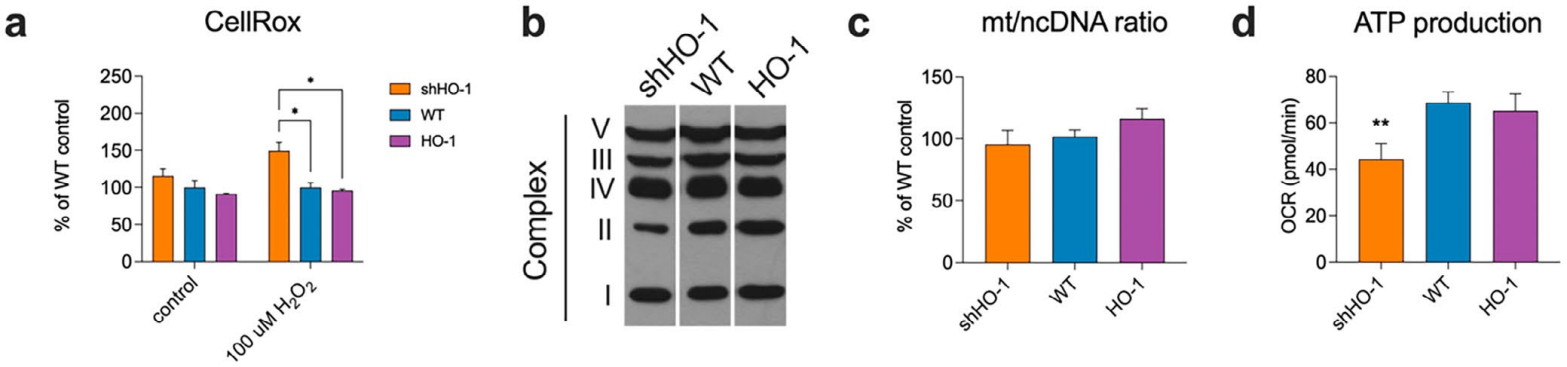
HO‐1 modulates oxidative stress and ATP production in B16‐F10 melanoma cells. (a) Flow cytometry analysis of cytoplasmic ROS levels upon 30‐min treatment with 100 μM H_2_O_2_ (each bar represents mean + SEM, *n* = 2, *p < 0.05). (b) Western blot analysis of mitochondrial respiratory chain complexes (representative blot, *n* = 2). (c) qRT‐PCR analysis of mitochondrial‐to‐nuclear DNA ratio (each bar represents mean + SEM, *n* = 4). (d) ATP production (each bar represents mean + SEM, *n* = 2–3, **p < 0.01).

Previous studies have shown that oxidative stress induced by H_2_O_2_ promotes melanogenesis in B16‐F10 cells by upregulating melanogenesis‐related genes and increasing mitochondrial ATP production. This ATP increase was attributed to elevated expression of ATP synthase in mitochondrial complex V (Kim and Lee 2013). Because HO‐1 is a key component of the NRF2‐driven oxidative stress response (Loboda et al. 2016), we next tested whether HO‐1 deficiency alters cytoplasmic ROS production, potentially affecting melanogenesis. As expected, ROS levels were elevated in H_2_O_2_‐treated melanoma cells with silenced HO‐1 (Figure 2a). However, under basal conditions, ROS levels did not differ significantly between the cell lines, suggesting that bulk steady state cytoplasmic ROS is unlikely to be sufficient to account for the HO‐1‐dependent pigmentation phenotype. However, these measurements do not rule out a contribution of compartmentalized or transient ROS signaling.

We also observed that the expression of mitochondrial complex V was comparable among cell lines with different HO‐1 status (Figure 2b). Moreover, no significant differences were detected in the mitochondrial‐to‐nuclear DNA ratio (Figure 2c), indicating that HO‐1 does not affect mitochondrial biogenesis. Metabolic analysis revealed that HO‐1 deficiency reduced ATP production rates (Figure 2d), which could potentially affect melanogenesis (Kim and Lee 2013). However, this was not the determining factor, as HO‐1 overexpression increased pigmentation and tyrosinase activity (Figure 1a,h) without altering ATP levels (Figure 2d).

### 3.3 HO‐1 Promotes Melanogenesis Under Pigment‐Inducing Conditions

**FIGURE 3:**
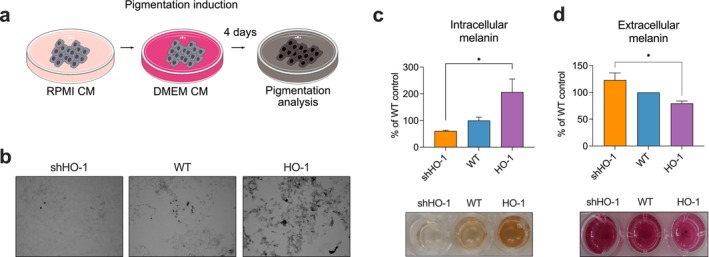
HO‐1 promotes induced melanogenesis in B16‐F10 melanoma cells. (a) Experimental scheme for melanogenesis induction. (b) Bright‐field microscopy images of melanin accumulation after 4 days of culture in DMEM CM (representative images, 100×). (c) Intracellular melanin content measured in cell lysates using spectrophotometric quantification (top graph, each bar represents mean + SEM; *p < 0.05, *n* = 3). Bottom panel shows picture of representative reaction wells. (d) Extracellular melanin content measured in cell media using spectrophotometric quantification (top graph, each bar represents mean + SEM; *p < 0.05, *n* = 3). Bottom panel shows picture of representative reaction wells.

To examine whether HO‐1 affects not only basal but also induced melanogenesis we stimulated pigmentation by culturing B16‐F10 melanoma cell lines for 4 days in DMEM complete medium (CM), which contains ~3.5‐fold more tyrosine (a melanogenesis substrate) than RPMI 1640 medium routinely used for B16‐F10 culture (Figure 3a) (Halaban et al. 2002, 2001). We observed that shHO‐1 melanoma cells exhibited markedly reduced melanin synthesis, microscopically and on intracellular melanin levels, compared with HO‐1‐overexpressing cells (Figure 3b,c). In contrast, when melanin was measured in the culture media, the pattern was reversed (Figure 3d). Taken together, these results demonstrate that HO‐1 is required for efficient melanogenesis in the B16‐F10 cell line, where its deficiency reduces tyrosinase activity and melanin synthesis but enhances melanin export from melanoma cells.

### 3.4 HO‐1 Deficiency in Melanoma Cells Affects Melanosome Abundance and Trafficking

**FIGURE 4:**
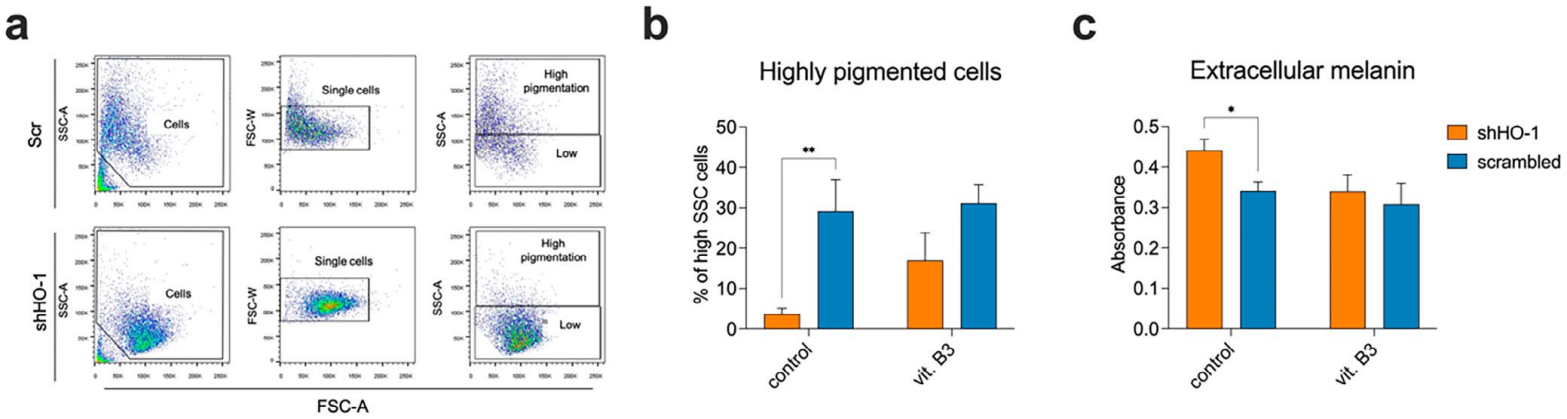
HO‐1 deficiency alters melanosome content and trafficking in B16‐F10 melanoma cells. (a) Flow cytometry analysis (FSC/SSC) of cell size and granularity; high granularity indicates high melanosome content. Representative dot plots. (b) Melanin content measured as % of SSC^high^ cells after 4‐day melanogenesis induction with or without 10 μM vitamin B3 (each bar represents mean + SEM, *n* = 3, **p < 0.01). (c) Spectrophotometric quantification of extracellular melanin content in cell culture media from cells treated with 10 μM vitamin B3 (each bar represents mean + SEM, *n* = 3, *p < 0.05).

Next, we analyzed B16‐F10 cell granularity as a proxy for melanosome content (Bajpai et al. 2023). Flow cytometry revealed that shHO‐1 cells exhibited reduced granularity, suggesting that their lower melanin levels may be at least partially explained by decreased intracellular melanosome content (Figure 4a). To determine whether this effect was associated with enhanced melanosome secretion, we treated cells with niacinamide (vitamin B3, 10 μM), which inhibits melanosome transfer without affecting melanogenic enzyme activities (Hakozaki et al. 2002). The results showed that inhibition of melanosome secretion increased the proportion of SSC^high^ cells in shHO‐1 melanoma cells but not in controls (Figure 4b). Concurrently, niacinamide reduced extracellular melanin release from shHO‐1 cells to levels comparable to those of control cells (Figure 4c). Taken together, these observations indicate that HO‐1 modulates melanoma cell pigmentation by influencing both melanin synthesis and melanosome trafficking.

### 3.5 Stromal HO‐1 Affects Melanoma Pigmentation

**FIGURE 5:**
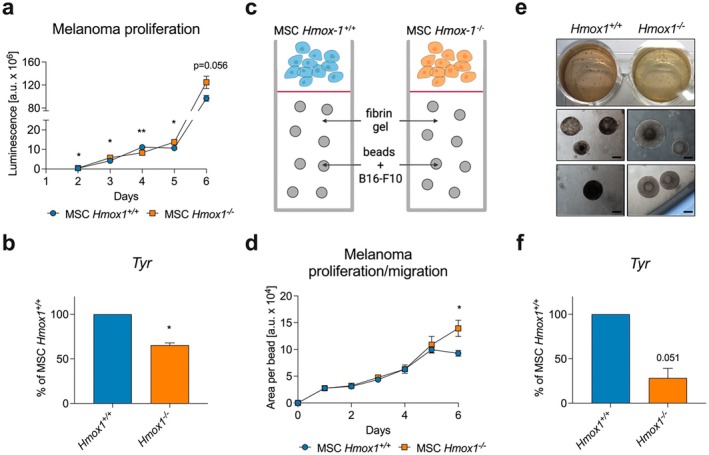
Stromal HO‐1 modulates melanoma pigmentation. (a) Proliferation of B16‐F10‐Luc cells during co‐culture with *Hmox1* ^+/+^ and *Hmox1* ^−/−^ MSCs, measured using IVIS Lumina imaging system (each bar represents mean ± SEM, *n* = 4, representing MSC isolations from 4 separate mice per genotype, *p < 0.05, **p < 0.01). (b) qRT‐PCR analysis of *Tyr* expression in melanoma cells after 6 days of co‐culture with MSCs of different genotypes; values normalized to *Hmox1* ^+/+^ MSC co‐cultures (each bar represents mean + SEM, *n* = 2, *p < 0.05). (c) Experimental scheme of the fibrin bead assay with B16‐F10 melanoma cells and *Hmox1* ^+/+^ or *Hmox1* ^−/−^ MSCs. (d) Growth dynamic of melanoma spheres in the fibrin gel, quantified using ImageJ (each bar represents mean ± SEM, *n* = 4, *p < 0.05). (e) Representative macroscopic (top) and microscopic (bottom) images of melanoma spheres after 6 days of fibrin bead co‐culture. (f) qRT‐PCR analysis of *Tyr* expression in B16‐F10 melanoma cells after 6 days of fibrin bead co‐culture with *Hmox1* ^+/+^ or *Hmox1* ^−/−^ MSCs; values normalized to *Hmox1* ^*+/+*^ MSCs co‐culture (each bar represents mean + SEM, *n* = 4).

We previously demonstrated that HO‐1 levels in host stromal cells influence melanoma growth in mice (Was et al. 2020). To test whether stromal HO‐1 affects melanoma pigmentation we co‐cultured B16‐F10 melanoma cells with GFP‐positive mesenchymal stromal cells (MSCs) isolated from *Hmox1* ^*+/+*^ or *Hmox1* ^*−/−*^ mice. Both MSC genotypes supported comparable melanoma cell growth, as shown by similar luminescence of B16‐F10‐Luc cells over 6 days (Figure 5a). After co‐culture, GFP‐negative melanoma cells were sorted by FACS and analyzed for *Tyr* mRNA expression, which was reduced in cells grown with *Hmox1* ^*−/−*^ MSCs (Figure 5b). These data indicate that stromal HO‐1 may promote melanogenesis in melanoma cells by regulating tyrosinase gene expression rather than enzymatic activity, as observed for cancer‐cell‐intrinsic HO‐1.

To determine whether this effect required direct cell–cell contact, we used a fibrin gel bead assay in which B16‐F10‐coated latex beads were embedded in a fibrin matrix and co‐cultured with *Hmox1* ^*+/+*^ or *Hmox1* ^*−/−*^ MSCs, preventing physical interaction (Figure 5c). Melanoma growth remained comparable between conditions; however, after 6 days, melanoma spheres co‐cultured with *Hmox1* ^*−/−*^ MSCs continued to expand, whereas those with *Hmox1* ^*+/+*^ MSCs ceased growth (Figure 5d). Notably, spheres exposed to *Hmox1* ^*−/−*^ MSCs appeared less pigmented, as assessed by macroscopic appearance and microscopy (Figure 5e), and showed a trend toward reduced *Tyr* expression (*p* = 0.051) (Figure 5f). Collectively, these findings suggest that stromal HO‐1 deficiency diminishes melanoma pigmentation through soluble mediators or changes in the available nutrients, as the effect occurred independently of direct MSC‐melanoma contact.

### 3.6 Associations of HO‐1 With Pigmentation Pathways in Human Melanoma Transcriptomes

**FIGURE 6:**
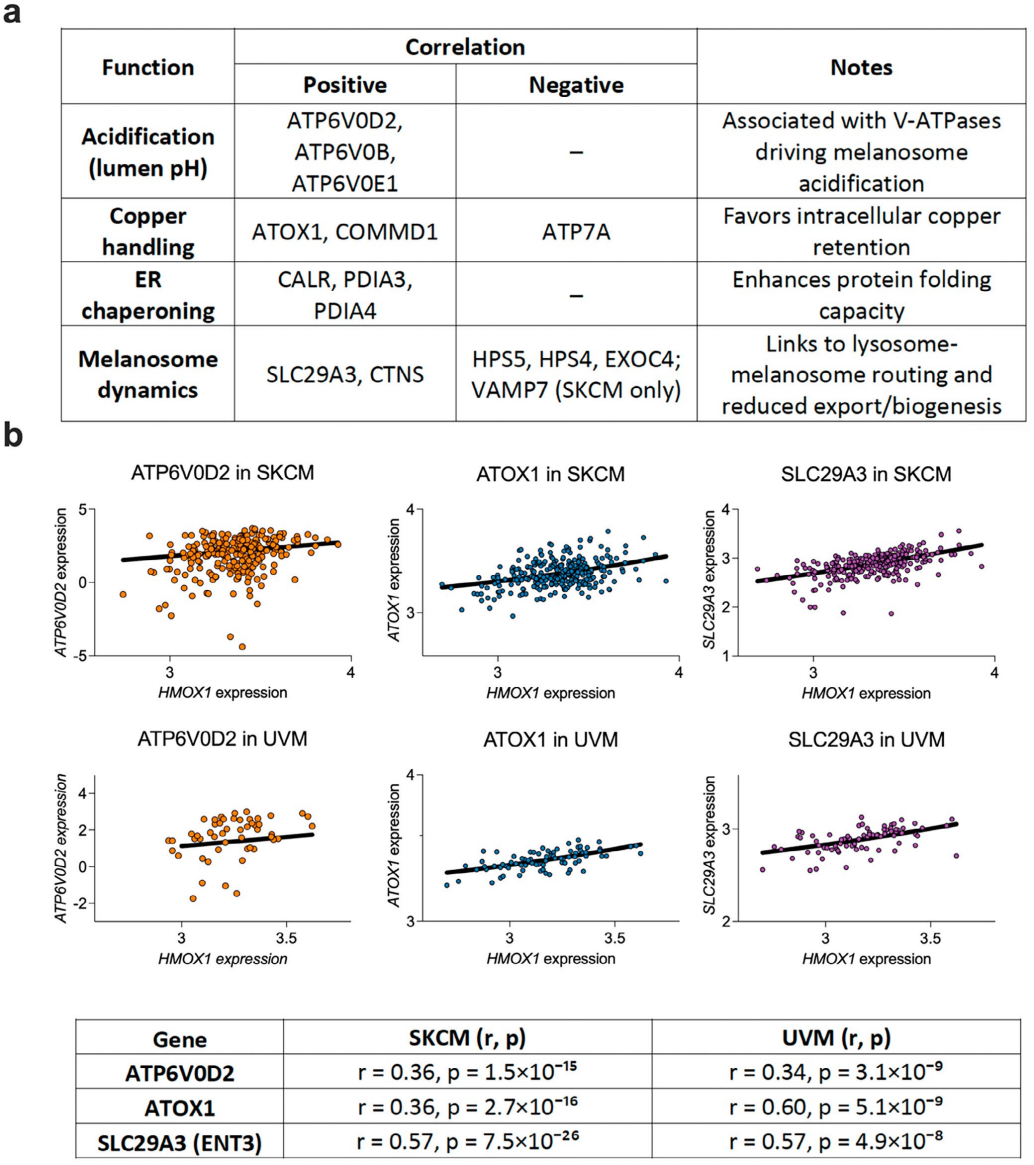
Correlation of *HMOX1* expression with pigmentation‐associated pathways in skin cutaneous melanoma (SKCM) and uveal melanoma (UVM). (a) Summary of significant correlations from the TCGA Firehose Legacy datasets (SKCM and UVM). (b) Representative scatterplots showing correlations between *HMOX1* and selected genes in SKCM (top) and UVM (bottom): *ATP6V0D2* (melanosome acidification), *ATOX1* (copper handling), and *SLC29A3* (lysosome–melanosome crosstalk), using mRNA expression (RNA Seq V2 RSEM) with both axes displayed on a log_2_ scale. Lower table contains Pearson correlation coefficients and *p*‐values for the selected correlations.

Building on our finding that HO‐1 modulates pigmentation in murine melanoma cells, we next explored whether *HMOX1* expression correlates with pigmentation‐associated pathways in human melanomas. Using a literature‐based list (Baxter et al. 2019) and expert‐curated list of genes associated with pigmentation and trafficking (Table S2), we analyzed the skin cutaneous melanoma transcriptomes (SKCM, TCGA Firehose Legacy). *HMOX1* expression was positively associated with pigmentation‐related modules, particularly genes involved in melanosome acidification (e.g., *ATP6V0D2*, *ATP6V0B*) (Pietrement et al. 2006; Tabata et al. 2008; Nuckels et al. 2009), copper chaperoning genes (*ATOX1*, *COMMD1*), which deliver copper ions essential for tyrosinase function (Bajpai et al. 2023; Pretzler and Rompel 2024; Hamza et al. 2003; Phillips‐Krawczak et al. 2014) and ER protein folding (*CALR, PDIA3*) (Jimbow et al. 2000; Lam and Lim 2021; Halaban et al. 2000)—all essential for proper melanosomal enzyme activity and maturation (Figure 6a,b). Similar correlations were observed in the uveal melanoma dataset (UVM, TCGA Firehose Legacy).

Conversely, *HMOX1* was negatively correlated with genes mediating vesicle trafficking and melanosome biogenesis (e.g., *VAMP7*, *EXOC4*, *HPS4*, *HPS5*) (Dennis et al. 2016; Tarafder et al. 2014; Moreiras et al. 2020; Helip‐Wooley et al. 2007; Suzuki et al. 2002), consistent with (but not proving) a transcriptional pattern that may reflect increased intracellular pigment retention and/or reduced export. Notably, *HMOX1* showed strong association with lysosomal transporters such as *SLC29A3*, implicated in disorders featuring hyperpigmentation (Cliffe et al. 2009; Priya et al. 2010). By contrast, correlations with core melanogenic enzymes (*TYR*, *TYRP1*, *DCT*, *MLANA*) were weak or absent (Table S2). Together, these findings identify coordinated organelle‐related gene programs that covary with *HMOX1* expression in human melanoma, with the strongest associations involving melanosome/lysosome function and metal handling rather than canonical melanogenic enzyme transcripts, providing clinical context consistent with our functional observations in the B16‐F10 model.

To assess whether these melanoma cohort gene signatures correspond to transcript changes in our experimental model, we performed targeted qRT–PCR of selected candidates (*Atox1*, *Commd1*, *Slc29a3*, and *Ctns*) in B16‐F10 WT and HO‐1 cells and did not detect significant differences (Figure S2). This suggests that the TCGA covariation with HMOX1 does not necessarily translate into direct transcript changes for these individual genes in our B16‐F10 system.

### 3.7 HO‐1 Expression Is Dispensable for Melanocyte Differentiation

**FIGURE 7:**
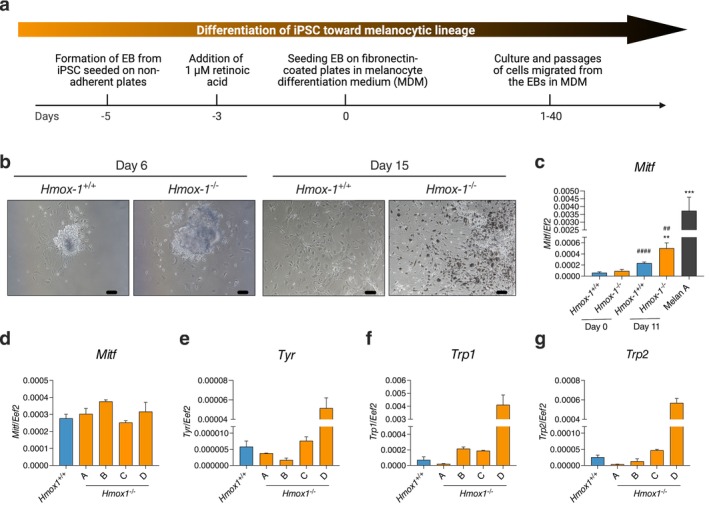
HO‐1 status does not affect iPSC differentiation toward melanocytes. (a) Timeline of the melanocyte differentiation protocol. (b) Phase‐contrast microscopic images of cells migrating from EBs at Days 6 and 15 of differentiation (representative images, 100×). (c) qRT‐PCR analysis of *Mitf* expression at Day 0 and at Day 11 of differentiation compared with Melan‐A melanocytes; *Eef2* was used as a housekeeping gene (each bar represents mean + SEM, *n* = 3–4, **p < 0.01 vs. *Hmox1* ^+/+^;***p < 0.001 Melan A vs. *Hmox1* ^−/−^ at Day 0; ^##^ *p* < 0.01 vs. Day 0; ^####^ *p* < 0.0001 vs. Day 0). (d–g) qPCR analysis of melanocyte markers at Day 40 of differentiation, in *Hmox1* ^+/+^ and four independent *Hmox1* ^−/−^ iPSC‐derived lines; *Eef2* was used as a housekeeping gene (each bar represents mean + SEM).

Since HO‐1 silencing reduced pigmentation in B16‐F10 melanoma cells, we next examined HO‐1 function in non‐malignant melanocytic contexts, focusing first on melanocytic differentiation from murine iPSCs. iPSCs were generated from tail‐tip fibroblasts of *Hmox1* ^*+/+*^ and *Hmox1* ^*−/−*^ mice (Figure S3a–e) and differentiated into the melanocytic lineage (Figure 7a). Following the protocol of Yang et al. (2011), we differentiated cells for up to 40 days, with cells migrating out from embryoid bodies (EBs) as early as on Day 6 of differentiation (Figure 7b).

By Day 11, *Mitf* expression increased in both genotypes but was more strongly induced in *Hmox1* ^*−/−*^ cells (Figure 7c), coinciding with the appearance of small pigmented clusters in some *Hmox1* ^*−/−*^ cultures by Day 15 (Figure 7b). However, *Mitf* levels remained far below those in mature Melan‐A melanocytes (Figure 7c), and by Day 40, expression was comparable across *Hmox1* ^*+/+*^ and four independent *Hmox1* ^*−/−*^ iPSC‐derived lines (Figure 7d). At this stage, tyrosinase, a key MITF target, was only weakly expressed and varied considerably between independent iPSC‐derived cell lines (Figure 7e). Similar variability was seen for other MITF targets, *Trp1* and *Trp2* (Figure 7f,g), likely reflecting stochastic differences in maturation rather than HO‐1‐dependent effects. This heterogeneity can be attributed to clonal and reprogramming‐related variability of iPSCs. Collectively, these data indicate that HO‐1 is dispensable for iPSC differentiation into melanocytes.

### 3.8 HO‐1 Expression Does Not Affect Pigmentation of Mature Melanocytes

**FIGURE 8:**
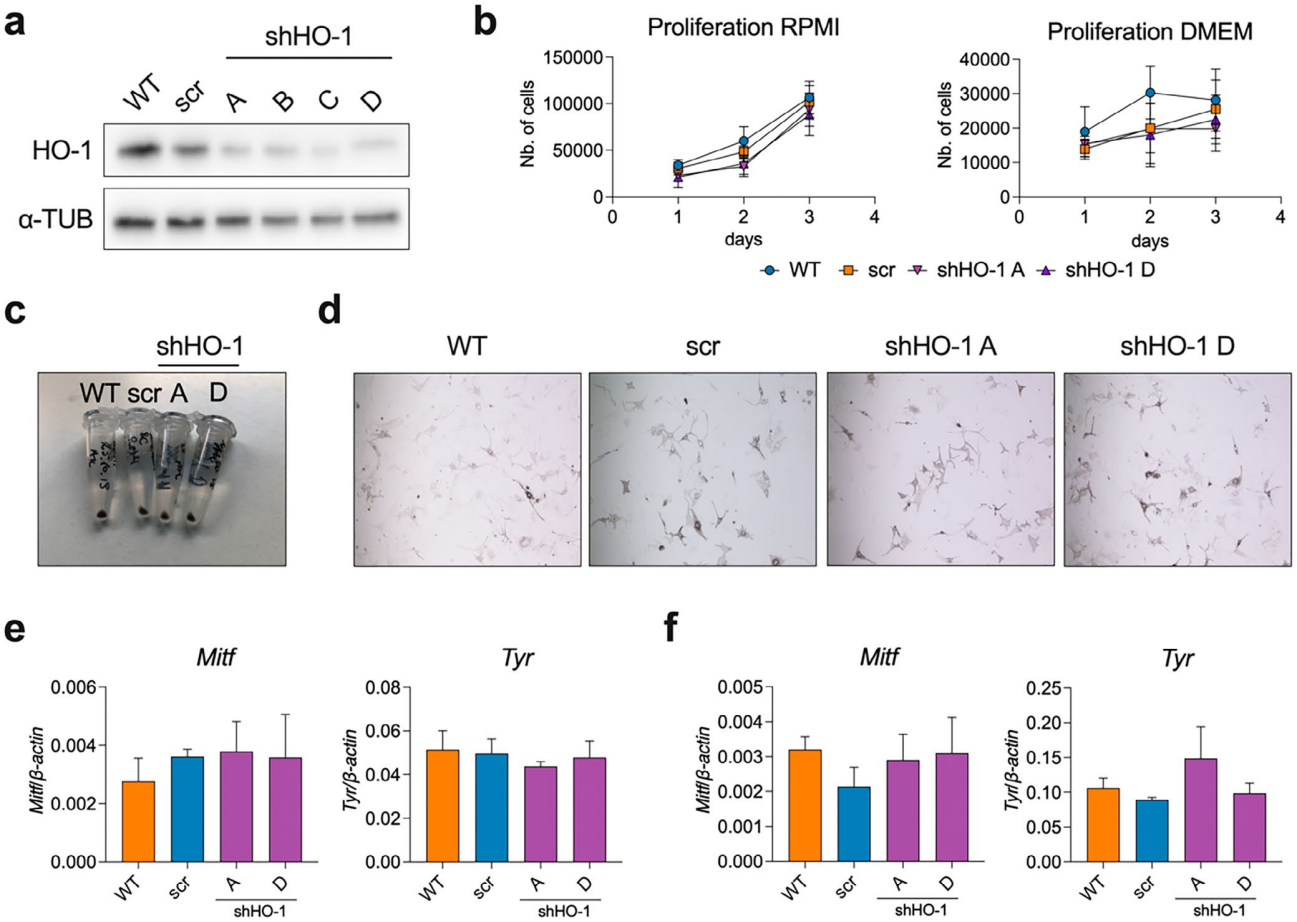
HO‐1 knockdown does not affect pigmentation in Melan‐A murine melanocytes. (a) Western blot analysis of HO‐1 protein expression in untreated control (WT), scrambled control (scr) and four shHO‐1‐transduced cell lines (shHO‐1, A–D); tubulin was used as a loading control (representative blot). (b) Proliferation of WT, scr, and two shHO‐1 cell lines (A, D) measured with Muse Cell Analyzer (each bar represents mean ± SEM, *n* = 3). (c) Representative image of cell pellets of WT, scr, and shHO‐1 (A, D) Melan‐A cells cultured in RPMI 1640 for 48 h. (d) Bright‐field microscopy images of Melan‐A cell lines cultured in DMEM for 48 h (representative pictures, 100×). (e) qRT‐PCR analysis of *Mitf* and *Tyr* in cells cultured in RPMI 1640. (f) qRT‐PCR analysis of *Mitf* and *Tyr* in cells cultured in DMEM for 48 h; *β*‐Actin (*Actb*) served as a housekeeping gene (each bar represents mean ± SEM, *n* = 3).

The role of HO‐1 in mature melanocytes was examined using the immortalized murine melanocyte line Melan‐A, in which HO‐1‐deficient cells were generated by retroviral shHO‐1 transduction. All four shRNA constructs (A–D) efficiently reduced HO‐1 protein levels (Figure 8a); subsequent analyzes focused on shHO‐1 A and D. Cells were cultured in RPMI 1640 CM to maintain proliferation or in DMEM CM to induce melanogenesis. HO‐1 knockdown did not affect Melan‐A proliferation (Figure 8b) or survival (Figure S4). In contrast to melanoma cells (Figure 1a), HO‐1 deficiency did not alter pigmentation, as demonstrated by unchanged pellet color (Figure 8c) and identical microscopic appearance after melanogenesis induction (Figure 8d). Consistently, HO‐1 status had no effect on *Mitf* or *Tyr* expression in either RPMI 1640 (Figure 8e) or in pro‐melanogenic DMEM CM (Figure 8f) cultures. Together, these results indicate that, unlike in B16‐F10 melanoma cells, HO‐1 knockdown does not alter pigmentation in the Melan‐A melanocyte model under our experimental conditions.

## 4 Discussion

Melanin contributes to melanoma aggressiveness and therapy resistance (Brożyna et al. 2013). Here, we demonstrate that HO‐1, a stress‐responsive enzyme frequently upregulated in melanoma (Okamoto et al. 2006), modulates melanogenesis. In genetically modified B16‐F10 melanoma cells, we observed that pigmentation levels directly reflected HO‐1 expression. HO‐1 was inhibited via shRNA‐mediated silencing, which avoids the off‐target effects of metalloporphyrin inhibitors (e.g., SnPPIX, ZnPPIX) that can impact melanogenic enzymes independently of HO‐1 (Guo et al. 2023) or even induce HO‐1 expression (Mucha et al. 2018). Although the underlying mechanism remains to be fully elucidated, HO‐1 depletion did not alter the expression of melanogenesis genes but instead reduced tyrosinase enzymatic activity. To our knowledge, this is the first evidence that HO‐1 modulates tyrosinase activity without affecting transcription of canonical melanogenic regulators.

Cellular pigmentation depends on both melanin synthesis and controlled melanosome export. Our results suggest that HO‐1 regulates both arms of this equilibrium. HO‐1‐depleted melanoma cells exhibited reduced tyrosinase activity and enhanced melanosome secretion, as indicated by lower cell granularity and increased levels of extracellular melanin. This increase in melanosome export was reversed by niacinamide, a melanosome‐transfer inhibitor (Hakozaki et al. 2002). Thus, HO‐1 increases pigmentation by promoting melanin synthesis and restraining its export. In line with this notion, transcriptomic analyses of publicly available datasets on uveal and SKCM patient cohorts show that *HMOX1* expression is associated with genes involved in melanosome maturation and copper chaperoning, and negatively with genes controlling export and trafficking pathways. As these are bulk‐tumor correlations, these analyzes are affected not only by tumor cells transcriptome but also by stromal and immune cells present in the tumor microenvironment. Therefore, the correlations should be interpreted as associations rather than evidence of causality. Nonetheless, the coordinated enrichment of pigmentation‐related organelle programs in HMOX1‐high tumors provides clinical context consistent with our functional observations in melanoma cells. Differences between bulk patient correlations and a single murine cell‐line perturbation likely reflect tumor heterogeneity and microenvironmental contributions in vivo, as well as the possibility that HO‐1 impacts pigmentation predominantly through posttranscriptional mechanisms in our model. Together, these findings support a context‐dependent link between HO‐1 and pigmentation in melanoma.

HO‐1‐dependent effects on melanin synthesis and pigment handling were most evident in the malignant and disease‐associated context. A similar disturbance in pigment retention is also observed in OCA4 melanocytes carrying the *underwhite* mutation, which also show excessive melanosome export and hypopigmentation (Costin et al. 2003). However, in a non‐malignant background, including *Hmox1* ^+/+^ and *Hmox1* ^−/−^ iPSCs, HO‐1 is dispensable for proper melanocyte maturation, although it may influence differentiation kinetics. Pigmentation and melanogenesis gene expression increase during differentiation regardless of HO‐1 status. Furthermore, in Melan‐A melanocytes, HO‐1 status does not influence pigmentation, proliferation, or viability. Thus, in the models examined here, HO‐1‐dependent pigmentation regulation was robust in melanoma cells but not detected in iPSC‐derived melanocytes during differentiation or in Melan‐A melanocytes, supporting a context‐dependent effect. Differences in differentiation state, metabolic state, epigenetic landscape, and culture conditions between these systems likely contribute to this divergent HO‐1 dependency.

Melanoma cells have increased levels of oxidative stress compared to normal melanocytes. This reflects metabolic rewiring, high melanin turnover, and exposure to oncogenic and microenvironmental stimuli (Arslanbaeva and Santoro 2020). Our previous work demonstrated that HO‐1 overexpression in melanoma cells confers protection against H_2_O_2_‐induced oxidative stress (Was et al. 2006). However, ROS production, although classically linked to downregulation of melanogenic enzymes via MITF‐dependent mechanisms (Jiménez‐Cervantes et al. 2001), did not differ by HO‐1 status under basal conditions, suggesting that differences in steady state cytoplasmic ROS are unlikely to be the sole driver of the pigmentation phenotype. This context dependency likely reflects the distinct oxidative, metabolic, and transcriptional landscape of melanoma compared to normal melanocytes.

Cellular ATP availability modulates MITF‐dependent transcriptional programs to regulate melanogenesis (Kim and Lee 2013). However, in our study, ATP availability does not appear to be the primary determinant of melanogenesis, as the stronger pigmentation of HO‐1‐overexpressing cells was not accompanied by an increase in ATP production compared with wild‐type controls. Signaling modulators such as PARP1 (Choi et al. 2017) and p53 (Lim et al. 2016; Cui et al. 2007), both known to modulate MITF and tyrosinase, are strongly influenced by HO‐1 (Chudy et al. 2024). However, in our model, HO‐1 status did not alter MITF or tyrosinase on transcriptional or protein levels. Taken together, these results indicate that neither ATP availability nor canonical MITF signaling adequately explain the HO‐1‐dependent pigmentation phenotype.

A more mechanistically relevant aspect of HO‐1 function in melanoma may lie in regulating the intracellular iron pool. As the enzymatic degradation of heme by HO‐1 releases ferrous iron (Fe^2+^), the enzyme directly contributes to cellular iron homeostasis (de Oliveira et al. 2022). Reduced iron levels have been reported, among others, in HO‐1‐depleted RAW264.7 macrophages (Mitterstiller et al. 2016). Iron ions have been implicated in promoting pigmentation (Palumbo et al. 1985); and higher iron contents have been observed in melanosomes of melanoma cells and dysplastic naevi compared to healthy melanocytes (Pavel et al. 2004). Consistently, in melanoma, reduced intracellular Fe^2+^ levels have been associated with decreased expression of iron‐dependent oxidative phosphorylation complexes (including complex II) and with a diminished oxygen consumption rate (OCR) (Rizzollo et al. 2025). These observations support a model in which HO‐1 enhances tyrosinase function and melanosome maturation partly through maintaining iron availability and iron‐dependent mitochondrial activity. Future studies defining how HO‐1‐derived iron influences melanosome pH, copper loading of tyrosinase, or melanosomal redox homeostasis may further clarify this connection.

Beyond cell‐intrinsic mechanisms, the tumor stroma critically influences melanoma biology (Ju et al. 2018). Indeed, HO‐1 expressed by tumor‐associated macrophages and stromal cells impacts tumor progression and pigmentation (Torisu‐itakura et al. 2000). In our co‐culture models, HO‐1‐deficient MSCs reduced melanoma pigmentation, even in the absence of direct cell contact, indicating involvement of as‐yet‐unidentified paracrine signaling. IL‐6, IL‐1α, and TNF‐α can mediate paracrine inhibition of melanogenesis (Swope et al. 1991), while TGF‐β1 decreases tyrosinase and TRP‐1 levels in B16‐F10 melanoma cells (Hwang et al. 2014). However, cytokine profiling excluded HO‐1‐dependent differences in IL‐6, IL‐1α, TNF‐α, or TGF‐β1 secretion between our MSC genotypes (Nowak et al. 2018). Alternatively, the observed effects might be connected to changes in available nutrients, but the exact mechanisms need further investigation.

This study has several limitations that should be considered when interpreting the data. First, the melanocytic systems used here (B16‐F10 melanoma cells, Melan‐A melanocytes, and iPSC‐derived melanocytes) represent distinct cell states and are maintained in different culture conditions, which are known to influence pigmentation. Accordingly, our conclusions are restricted to these experimental conditions and should not be interpreted as a one‐to‐one comparison between non‐malignant melanocytic models and melanoma. Second, although our findings support a role for HO‐1 in regulating melanogenesis through altered tyrosinase activity and pigment handling, the relative contribution of transcriptional versus posttranscriptional mechanisms remains incompletely resolved, and broader assessment of melanosome acidification, metal‐handling and lysosomal pathways at the protein and functional level will be required. Third, although bulk basal cytosolic ROS levels did not differ by HO‐1 status, this does not exclude compartmentalized or transient redox effects, and direct testing of redox involvement will require targeted perturbation approaches (e.g., antioxidant buffering or ROS modulation under matched melanogenesis‐inducing conditions) coupled to pigmentation and tyrosinase readouts. Finally, associations between *HMOX1* expression and pigmentation‐related programs in bulk melanoma transcriptomes provide clinical context but do not establish a causal relationship and may be influenced by tumor heterogeneity and microenvironmental composition. In line with this, candidate genes identified in patient datasets did not show concordant mRNA changes in our B16‐F10 model, indicating that cohort‐level pathway associations may not translate directly to individual experimental systems. Future validations in human melanoma models and parallel analysis of normal skin datasets will help determine how well our conclusions extend to human melanocytic biology.

In summary, we demonstrated that HO‐1 influences melanogenesis in melanoma cells but is largely dispensable for pigmentation of normal melanocytes. Reanalysis of patient transcriptomic datasets provides supporting clinical context, revealing associations between *HMOX1* expression and pigmentation‐related pathways in human melanomas. Given the established link between pigmentation, immune evasion, and therapeutic resistance, targeting HO‐1 may offer a strategy to modulate pigment‐associated tumor phenotypes in combination with existing treatments.

## Author Contributions

**Martin Petrovic:** investigation, writing – review and editing. **Grażyna Jamróg:** investigation, writing – review and editing. **Wolfgang Weninger:** writing – review and editing, resources. **Matthias Farlik:** writing – review and editing, funding acquisition, resources. **Jacek Stępniewski:** investigation, writing – review and editing, methodology. **Witold Nowak:** methodology, investigation. **Rościsław Krutyhołowa:** investigation, writing – review and editing. **Anna Tejchman‐Skrzyszewska:** investigation, methodology. **Milena Mazan:** investigation, methodology. **Halina Waś:** conceptualization, methodology, investigation, writing – review and editing. **Agnieszka Seretny:** conceptualization, methodology, investigation, formal analysis, data curation, writing – original draft, writing – review and editing, visualization, validation, software. **Maciej Cieśla:** investigation, writing – review and editing, methodology. **Alicja Józkowicz:** conceptualization, resources, project administration, writing – original draft, writing – review and editing, supervision, funding acquisition. **Anna Kusienicka:** conceptualization, methodology, investigation, funding acquisition, writing – original draft, validation, visualization, writing – review and editing, formal analysis, project administration, resources, supervision, data curation.

## Funding

Funding for this study was provided by the HARMONIA grant (2012/06/M/NZ1/00008) awarded to A.J., the ETIUDA 7 doctoral scholarship (2019/32/T/NZ3/00387) awarded to A.K. by the Polish National Science Centre, the APART‐MINT fellowship of the Austrian Academy of Science (project 12025) awarded to A.K., the Special Research Programme (SFB) of the Austrian Science Fund (FWF) (SFB F61‐03) awarded to M.F., and institutional support for young scientists from the Faculty of Biochemistry, Biophysics and Biotechnology at the Jagiellonian University.

## Conflicts of Interest

The authors declare no conflicts of interest.

## Supporting information


**Figure S1:** Expression of *Hmox1* in B16‐F10 cell lines. qRT‐PCR analysis of *Hmox1* in untreated control (WT), HO‐1 overexpressing (HO‐1), scrambled control (scr), and shHO‐1 transduced cells; *Eef2* was used as a housekeeping gene (each bar represents mean + SEM; *n* = 2. ***p < 0.001).
**Figure S2:** Targeted qRT–PCR of candidate genes linked to HMOX1‐associated modules in B16‐F10 WT and HO‐1 cells; *Actb* was used as a housekeeping gene (each bar represents mean + SEM; *n* = 3).
**Figure S3:** Generation and characterization of iPSCs derived from murine *Hmox1* ^+/+^ and *Hmox1* ^−/−^ fibroblasts. (a) Timeline of iPSC generation. (b) Phase‐contrast microscopy of iPSC morphology (representative pictures). (c) RT‐PCR analysis of pluripotency markers (*Sall4*, *Nanog*, *Rex1*). PCR products separated by agarose gel electrophoresis. (d) Pluripotency markers in iPSC lines assessed by alkaline phosphatase activity, and immunofluorescent stainings for CDy1 retention, OCT4, SSEA‐1, NANOG (representative images). (e). Spontaneous differentiation of iPSC towards three germ layers assessed by immunofluorescent staining: ectoderm (NFH and Nestin), mesoderm (Vimentin and αSMA), and endoderm (AFP and GATA4) (representative images).
**Figure S4:** Cell viability of untreated control (WT), scrambled control (scr) and shRNA‐transduced cell lines (shHO‐1 A and D) cultured in (a) RPMI or (b) DMEM at the indicated time points, measured with Muse Cell Analyzer (each bar represents mean + SEM; *n* = 3).

**Data S1:** Materials and methods for the iPSCs culture and differentiation.

**Table S1:** Contains details of cell culture media, oligos sequences and antibodies used in the study.

**Table S2:** Transcriptomic data analysis of pigmentation‐related gene sets. Normalized RNA‐seq data from the TCGA Skin Cutaneous Melanoma (SKCM) and Uveal Melanoma (UVM) cohorts (Firehose Legacy) were obtained via cBioPortal (https://www.cbioportal.org/).

## Data Availability

All data in the manuscript are available from the corresponding author upon request.
