# Supplementary material for: Heme Oxygenase‐1 Differentially Controls Pigmentation in Physiological and Pathological Melanogenesis
Source: Pigment Cell Melanoma Res. 2026 Jul 6;39(4):e70105. doi: 10.1111/pcmr.70105 (PMC13335822; doi:10.1111/pcmr.70105)
Supplement: Supplementary file 1 — Figure S1: Expression of Hmox1 in B16‐F10 cell lines. qRT‐PCR analysis of Hmox1 in untreated control (WT), HO‐1 overexpressing (HO‐1), scrambled control (scr), and shHO‐1 transduced cells; Eef2 was used as a housekeeping gene (each bar represents mean + SEM; n = 2. ***p < 0.001). Figure S2: Targeted qRT–PCR of candidate genes linked to HMOX1‐associated modules in B16‐F10 WT and HO‐1 cells; Actb was used as a housekeeping gene (each bar represents mean + SEM; n = 3). Figure S3: Generation and characterization of iPSCs derived from murine Hmox1 +/+ and Hmox1 −/− fibroblasts. (a) Timeline of iPSC generation. (b) Phase‐contrast microscopy of iPSC morphology (representative pictures). (c) RT‐PCR analysis of pluripotency markers (Sall4, Nanog, Rex1). PCR products separated by agarose gel electrophoresis. (d) Pluripotency markers in iPSC lines assessed by alkaline phosphatase activity, and immunofluorescent stainings for CDy1 retention, OCT4, SSEA‐1, NANOG (representative images). (e). Spontaneous differentiation of iPSC towards three germ layers assessed by immunofluorescent staining: ectoderm (NFH and Nestin), mesoderm (Vimentin and αSMA), and endoderm (AFP and GATA4) (representative images). Figure S4: Cell viability of untreated control (WT), scrambled control (scr) and shRNA‐transduced cell lines (shHO‐1 A and D) cultured in (a) RPMI or (b) DMEM at the indicated time points, measured with Muse Cell Analyzer (each bar represents mean + SEM; n = 3). [file PCMR-39-0-s004.docx]

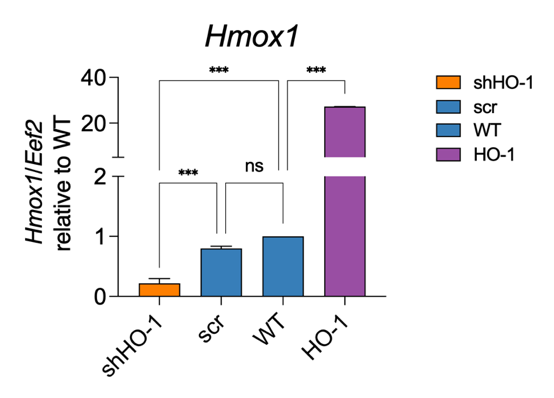


Figure S1. Expression of *Hmox1* in B16-F10 cell lines. qRT-PCR analysis of *Hmox1* in untreated control (WT), HO-1 overexpressing (HO-1), scrambled control (scr), and shHO-1 transduced cells; *Eef2* was used as a housekeeping gene (mean + SEM); n=2. *** - p<0.001.

Figure S2. Targeted qRT–PCR of candidate genes linked to HMOX1-associated modules in B16-F10 WT and HO-1 cells; *Actb* was used as a housekeeping gene (mean + SEM); n=3.


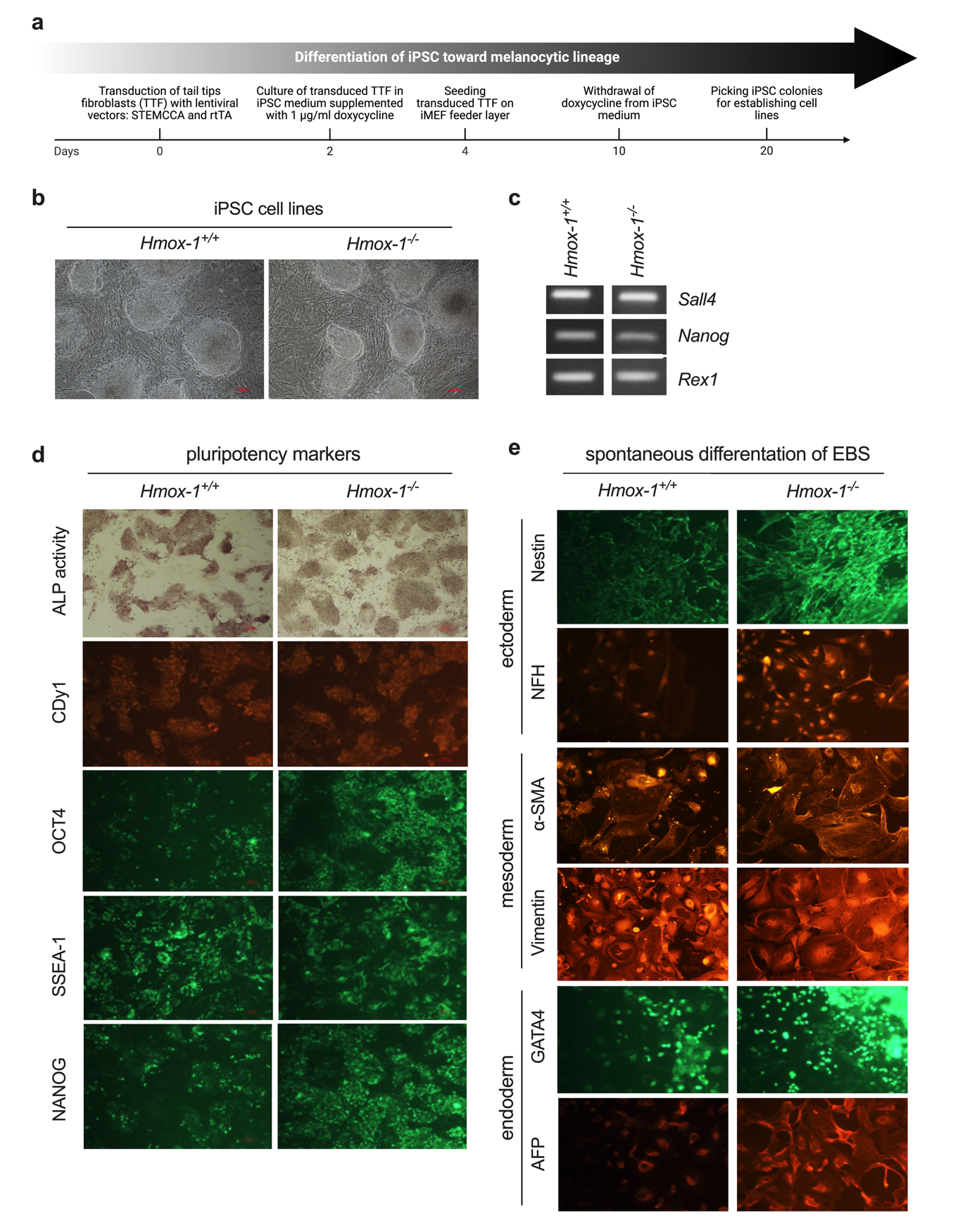


Figure S3. Generation and characterization of iPSCs derived from murine *Hmox1*^+/+^ and *Hmox1*^-/-^ fibroblasts. a. Timeline of iPSC generation. b. Phase-contrast microscopy of iPSC morphology. c. RT-PCR analysis of pluripotency markers (*Sall4*, *Nanog*, *Rex1*). PCR products separated by agarose gel electrophoresis. d. Pluripotency markers in iPSC lines assessed by alkaline phosphatase activity, and immunofluorescent stainings for CDy1 retention, OCT4, SSEA-1, NANOG; representative images. e. Spontaneous differentiation of iPSC towards three germ layers assessed by immunofluorescent staining: ectoderm (NFH and Nestin), mesoderm (Vimentin and αSMA), and endoderm (AFP and GATA4); representative images.


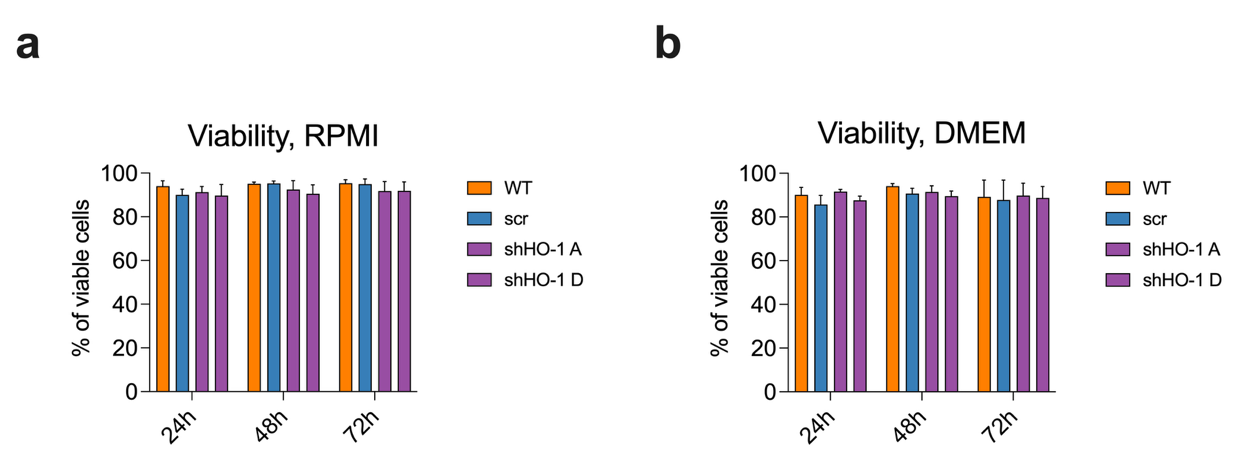
Figure S4. Cell viability of untreated control (WT), scrambled control (scr) and shRNA-transduced cell lines (shHO-1 A and D) cultured in (a) RPMI or (b) DMEM at the indicated time points, measured with Muse Cell Analyzer (each bar represents mean + SEM; n=3).
