# Supplementary material for: Heme Oxygenase‐1 Differentially Controls Pigmentation in Physiological and Pathological Melanogenesis
Source: Pigment Cell Melanoma Res. 2026 Jul 6;39(4):e70105. doi: 10.1111/pcmr.70105 (PMC13335822; doi:10.1111/pcmr.70105)
Supplement: Supplementary file 2 — Data S1: Materials and methods for the iPSCs culture and differentiation. [file PCMR-39-0-s003.docx]

**Generation of induced pluripotent stem cells (iPSCs).** Lentiviral vectors (LVs) for iPSC generation were produced in HEK293 cells transfected with psPAX2, pMD2.G, and pHAGE-STEMCCA plasmids (kindly provided by Dr. Gustavo Mostoslavsky, Boston University School of Medicine, USA) using polyethyleneimine (MW25000, Polysciences Inc.)^1^. Tail-tip fibroblasts (TTFs) from C57Bl6×FVB *Hmox1*^+/+^ and *Hmox1*^-/-^ mice were transduced with LVs-containing medium collected 48 hours post-transfection of HEK293 cells. After another 48 hours, the medium was replaced with iPSC medium supplemented with 1 μg/mL doxycycline (Sigma-Aldrich). On day 4 post-transduction, transduced TTFs were seeded onto a monolayer of mitotically inactivated murine embryonic fibroblasts (iMEFs), as described in Stepniewski et al.^2^ On day 10, doxycycline was withdrawn, and the cells were cultured for an additional 10 days in iPSC medium. Finally, individual iPSC colonies were transferred onto a monolayer of iMEFs.

**Generation of embryoid bodies (EBs).** To obtain pure iPSC lines and eliminate iMEFs, cells were seeded onto 0.1% gelatin-coated 6-well plates and incubated for 30 minutes at 37°C. Non-attached cells were collected, centrifuged, and seeded onto non-adherent plates. EBs were formed over 5 days in iPSC medium lacking mLIF and then transferred to 0.1% gelatin-coated 48-well plates. EB cultures were continued until cells began to migrate out of the aggregates. Immunofluorescent staining was performed to assess the lineage differentiation potential of iPSCs.

**Differentiation of iPSCs toward melanocytes.** Differentiation of iPSCs toward melanocytes was performed according to the protocol published by Yang et al.^3^ *Hmox1*^+/+^ and *Hmox1*^-/-^ iPSCs were seeded onto non-adherent culture dishes in iPSC medium lacking mLIF to form EBs. After 2 days, retinoic acid was added (final concentration: 1 μM; Sigma-Aldrich), and cultures were continued for an additional 3 days. EBs were then transferred to fibronectin-coated plates (10 ng/mL; Sigma-Aldrich) and cultured in melanocyte differentiation medium (MDM, Supplementary Table 1. ).

**Alkaline phosphatase (ALP) activity assay.** ALP activity^4^, a marker of pluripotent stem cells, was assessed using a colorimetric assay (Sigma-Aldrich) according to the manufacturer’s protocol. Stained cells were analyzed under an inverted microscope.

**CDy1 staining.** CDy1 is a fluorescent small molecule that selectively labels pluripotent stem cells^5^. iPSCs were incubated with 0.1 μg/mL CDy1 in iPSC medium for 1 hour at 37°C. The medium was then replaced with fresh iPSC medium, and the cells were incubated for an additional 2-3 hours at 37°C. Dye-retaining cells were visualized using fluorescence microscopy.

**Immunofluorescent staining.** Cells were fixed with 4% PFA for 10 minutes, washed with PBS and permeabilized with 0.1% Triton X-100 in PBS for 15 minutes. After three washes with PBS, samples were blocked with 4% bovine serum albumin (BSA) for 1 hour. After overnight incubation at 4°C with the respective primary antibody, cells were washed 5 times with PBS, and then incubated with secondary antibodies for 1 hour. The primary and secondary antibodies used in the study are included in Supplementary Table 1. Nuclei were counterstained with Hoechst 33342 (1 μg/mL; Sigma-Aldrich).

1. Sommer, C. A. *et al.* Induced Pluripotent Stem Cell Generation Using a Single Lentiviral Stem Cell Cassette. *Stem Cells* **27**, 543–549 (2009). DOI:10.1634/stemcells.2008-1075.

2. Stepniewski, J. *et al.* Induced pluripotent stem cells as a model for diabetes investigation. *Sci. Rep.* **5**, 1–14 (2015). DOI:10.1038/srep08597.

3. Yang, R. *et al.* Generation of Melanocytes from Induced Pluripotent Stem Cells. *J. Invest. Dermatol.* **131**, 2458–2466 (2011). DOI:10.1038/jid.2011.242.

4. Štefková, K., Procházková, J. & Pacherník, J. Alkaline Phosphatase in Stem Cells. *Stem Cells Int.* **2015**, 628368 (2015). DOI:https://doi.org/10.1155/2015/628368.

5. Kang, N.-Y., Yun, S.-W., Ha, H.-H., Park, S.-J. & Chang, Y.-T. Embryonic and induced pluripotent stem cell staining and sorting with the live-cell fluorescence imaging probe CDy1. *Nat. Protoc.* **6**, 1044–1052 (2011). DOI:10.1038/nprot.2011.350.
